# Fractionation of impulsive and compulsive trans-diagnostic phenotypes and their longitudinal associations

**DOI:** 10.1177/0004867419844325

**Published:** 2019-04-19

**Authors:** Samuel R Chamberlain, Jeggan Tiego, Leonardo F Fontenelle, Roxanne Hook, Linden Parkes, Rebecca Segrave, Tobias U Hauser, Ray J Dolan, Ian M Goodyer, Ed Bullmore, Jon E Grant, Murat Yücel

**Affiliations:** 1 Cambridge and Peterborough NHS Foundation Trust and Department of Psychiatry, University of Cambridge, Addenbrooke’s Hospital, Cambridge, UK; 2 Monash Institute of Cognitive and Clinical Neurosciences, School of Psychological Sciences, Monash University, Clayton, VIC, Australia; 3 The Max Planck UCL Centre for Computational Psychiatry and Ageing, University College London (UCL), London, UK; 4 Department of Psychiatry and Behavioural Neuroscience, University of Chicago, Chicago, IL, USA

**Keywords:** Impulsive, compulsive, phenotyping

## Abstract

**Objective:**

Young adulthood is a crucial neurodevelopmental period during which impulsive and compulsive problem behaviours commonly emerge. While traditionally considered diametrically opposed, impulsive and compulsive symptoms tend to co-occur. The objectives of this study were as follows: (a) to identify the optimal trans-diagnostic structural framework for measuring impulsive and compulsive problem behaviours, and (b) to use this optimal framework to identify common/distinct antecedents of these latent phenotypes.

**Method:**

In total, 654 young adults were recruited as part of the Neuroscience in Psychiatry Network, a population-based cohort in the United Kingdom. The optimal trans-diagnostic structural model capturing 33 types of impulsive and compulsive problem behaviours was identified. Baseline predictors of subsequent impulsive and compulsive trans-diagnostic phenotypes were characterised, along with cross-sectional associations, using partial least squares.

**Results:**

Current problem behaviours were optimally explained by a bi-factor model, which yielded dissociable measures of impulsivity and compulsivity, as well as a general disinhibition factor. Impulsive problem behaviours were significantly explained by prior antisocial and impulsive personality traits, male gender, general distress, perceived dysfunctional parenting and teasing/arguments within friendships. Compulsive problem behaviours were significantly explained by prior compulsive traits and female gender.

**Conclusion:**

This study demonstrates that trans-diagnostic phenotypes of 33 impulsive and compulsive problem behaviours are identifiable in young adults, utilising a bi-factor model based on responses to a single questionnaire. Furthermore, these phenotypes have different antecedents. The findings yield a new framework for fractionating impulsivity and compulsivity, and suggest different early intervention targets to avert emergence of problem behaviours. This framework may be useful for future biological and clinical dissection of impulsivity and compulsivity.

## Introduction

Young adults are particularly vulnerable to develop maladaptive, problem, behaviours (Spear, 2000). This vulnerability is thought to stem from neurodevelopmental alterations (Casey et al., 2019) coupled with concomitant changes in the environment (such as lessening parental oversight and increasing exposure to new peer groups). Two key concepts of particular relevance to understanding brain development and problem behaviours in young adulthood are ‘impulsivity’ and ‘compulsivity’ (Hollander et al., 2009). Impulsivity refers to behaviours that are unduly hasty and risky, and that lead to negative outcomes in the long term (Evenden, 1999). Examples of clinical disorders characterised by impulsivity include antisocial personality disorder, gambling disorder and substance use disorders (e.g. Krmpotich et al., 2015). Thus, an individual may undertake a spontaneous aggressive act (as in antisocial personality disorder), or may crave and consume a substance (or engage in a gambling opportunity), without due regard to potentially damaging consequences. Compulsivity, by contrast, refers to functionally impairing behaviours undertaken repeatedly, often according to rigid rules, manifesting classically in obsessive–compulsive disorder (OCD). Impulsive and compulsive problems are common in young adulthood, have negative effects on long-term outcomes and frequently co-occur (Black et al., 2017; Cerda et al., 2016; Fineberg et al., 2013).

Mechanistic, diagnostic and treatment progress in psychiatry has been hindered by an excessive focus on specific mental disorders, typically examined in isolation within clinical settings rather than a continuous or dimensional fashion in the population at large (Cuthbert and Insel, 2013). Intermediate phenotypes (trans-diagnostic markers) hold promise in this regard, and should be measurable along a continuum in the background population, existing in more extreme forms in people with conceptually related mental disorders. Recent reports from population-based studies have suggested that trans-diagnostic phenotypes are indeed likely (St Clair et al., 2017; Stochl et al., 2015). Thus, a crucial next step in impulsivity–compulsivity research and practice is to identify intermediate phenotypes that cut across related problem behaviours and that are measurable dimensionally both in the general population and in the context of existing categories of mental disorders.

Recent cross-sectional studies have shown that variation in a broad range of impulsive and compulsive behaviours is also present sub-clinically and that these behaviours can be grouped into latent dimensions of ‘impulsivity’ and ‘compulsivity’ (termed intermediate phenotypes), which are found to be positively correlated (Chamberlain et al., 2017; Guo et al., 2017). This positive correlation could not only reflect common underpinnings but also theoretically stem from common measurement bias (cross-talk across scale items due to item response bias). It is important to identify an optimal framework within which to conceptualise separable impulsive and compulsive problem behaviours as this could then be used to explore common or distinct antecedents. A recent resurgence of interest in bi-factor models in psychiatry (Reise, 2012; St Clair et al., 2017) has yet to be adequately deployed and tested in latent phenotyping studies of impulsivity and compulsivity (Chamberlain et al., 2017; Guo et al., 2017). Such bi-factor models incorporate a general factor (capturing common variance across all study measures), as well as specific factors, enabling – theoretically – these constructs to be truly fractionated statistically.

The aims of the current study were as follows: (a) to identify the optimal trans-diagnostic structural framework for measuring impulsive and compulsive problem behaviours (including consideration of a bi-factor model), and (b) to use this optimal framework to identify longitudinal antecedents of these latent phenotypes for the first time, with a particular focus on demographic, personality, parenting and friendship measures. We focused on these measures as they were expected to contribute to subsequent problems, based on prior evidence. The optimal conceptual framework was identified using a competing models approach, and relationships between trans-diagnostic phenotypes and other measures were elicited using the innovative statistical approach of partial least squares (PLS), which is well suited for data likely to be correlated and non-normally distributed (Abdi and Williams, 2013).

## Method

### Participants

We contacted all individuals from the Neuroscience in Psychiatry Network (NSPN), a longitudinal population-based cohort study examining brain development (Kiddle et al., 2017), via email. Participants completed an online survey implemented in SurveyMonkey in 2018 and had also provided baseline questionnaires via the post (2012–2014). The current data collection (2018) examined a broader range of impulsivity/compulsivity measures, some of which were not available at the time the NSPN cohort was conceived (Chamberlain and Grant, 2018; Guo et al., 2017). The study was approved by Research Ethics Committee and individuals provided informed consent. Participants were given a £15 voucher for taking part.

### Measures of interest

Questionnaires collected presently (2018) were designed to capture relevant demographic characteristics (age, gender, ethnic group, level of education), trans-diagnostic measures of problem behaviours and other relevant measures of impulsivity and compulsivity, plus overall quality of life. The outcome instrument of interest, used to examine underlying latent phenotypes of impulsive and compulsive problem behaviours, was the Impulsive–Compulsive Behaviours Checklist (ICBC; Guo et al., 2017), which is described in more detail below. Other measures of interest collected in the current data round (2018) and at participant recruitment (2012–2014) are described in Table 1. The latter included measures of impulsive–compulsive tendencies, but also parenting and friendships, which we expected may impact the ultimate expression of the latent phenotypes on the ICBC. In addition, the following demographic information was examined, which was completed by the participant’s parent or legal guardian at study baseline: any perinatal complications in proband (yes/no), any history of head trauma in proband (yes/no), family educational level (parent’s/guardian’s number of years’ education completed) and any current or previous emotional/behavioural/mental health problem in the proband (yes/no).

**Table 1.** Summary of instruments included in the study.

Instrument	Description	Outcome measure(s)
Measures collected in current data round		
BIS-11 Brief version (Stanford et al., 2016; Steinberg et al., 2013)	BIS-11 Brief version: The BIS is an extensively used scale used for the classic measurement of impulsivity. We used the eight-item version, which has been previously validated in several studies. The questionnaire asks about impulsive tendencies and behaviours; for each item, the individual indicates the extent to which it applies to them (not at all, a little, quite a lot or very much; scored 0–4 per item)	Total score
Antisocial personality disorder symptoms (American Psychiatric Association, 2013)	Antisocial personality disorder symptoms were quantified using a tick-list of *DSM-5* criteria. For each symptom criterion, participants indicated whether the item applied to them or not	Total number of criteria endorsed
Padua Obsessive–Compulsive Inventory (Washington State University–Revision version; Burns et al., 1996; Sanavio, 1988)	This is a 39-item questionnaire measuring obsessive–compulsive symptoms in normative and clinical settings	Symptom domain scores (per Burns et al., 1996)
CHI-T (Chamberlain and Grant, 2018)	This is a 15-item scale capturing day-to-day aspects of compulsivity. For each item, the individual indicates whether the statement (e.g. ‘I hate leaving a task unfinished’) applies to them. Response options are as follows: *strongly disagree* (0), *disagree* (1), *agree* (2) or *strongly agree* (3)	Total score
OBQ Short Form (Moulding et al., 2011)	This is a 20-item short version of a widely used questionnaire to assess obsessional beliefs in the context of OCD. For each item, the person indicates how much they agree with a given statement, ranging from *disagree very much* (scored 1) to *agree very much* (scored 7)	Summary scores: Threat perception, perfection/intolerance of uncertainty, inflated responsibility and importance/control of thoughts
OCPD traits (American Psychiatric Association, 2013; Sheehan et al., 1998)	The diagnostic screening questions from the MINI were used for OCPD traits. For each item, participants were asked whether the statement applied to them, taking into consideration not only their own view but also what they felt other people (close associates) would think	Total number of OCPD traits endorsed
General psychopathology (depression/anxiety) from the MINI (Sheehan et al., 1998)	General psychopathology (depression/anxiety): The diagnostic screening questions from the MINI were used for depressive and anxiety disorders	Presence or depressive and/or anxiety symptoms (total items endorsed)
BBQ (Lindner et al., 2016)	The BBQ is a previously validated self-report quality of life scale, which covers six life areas (leisure time, view of life, creativity, learning, friends/friendship and view of self) that are important determinants of overall quality of life. This scale has been validated in various normative and clinical population settings	Total quality of life score
Measures collected at study baseline (2012–2014)		
BIS 11 full version (Patton et al., 1995)	This questionnaire asks about impulsive tendencies and behaviours; for each, the individual indicates the extent to which it applies to them (not at all, a little, quite a lot or very much; scored 0–4 per item)	Total score
The APSD (Munoz and Frick, 2007)	This is a 20-item questionnaire developed to assess psychopathy in young people. For each item, the individual indicates whether a given statement (e.g. ‘You engage in illegal activities’) is *not true at all* (0), *sometimes true* (1) or *definitely true* (2)	Total score
Padua Obsessive–Compulsive Inventory (Washington State University–Revision version; Burns et al., 1996; Sanavio, 1988)	Per current data round	Per current data round
General psychopathology (K10; Kessler et al., 2002)	The K10 was developed for the assessment of generalised psychological distress, including distress arising from anxiety and depression symptoms. There are 10 items each asking about experiences over the preceding 30 days. For each item, responses are as follows: *none of the time* (0), *a little of the time* (1), *some of the time* (2), *most of the time* (3) or *all of the time* (4)	Total score
FAD (Ridenour et al., 1999), PPQ, APQ-Child Short Form (Elgar et al., 2007) and MOPS (Parker et al., 1997)	Several complementary questionnaires relating to parenting approaches were included. The reader is referred to the cited manuscripts for more detailed descriptions of these instruments, except for the PPQ, which was developed bespoke for the NSPN study. The PPQ asks the extent to which each of 26 positively worded items related to one’s family when living at home (e.g. ‘We spent quality time together’; ‘If I was angry, I was still listened to’.) Each item is responded to *with always* (scored 0), *mostly* (scored 1), *sometimes* (scored 2) or *rarely* (scored 3). EFA was used to identify the optimal data structure across all these questionnaires’ items considered collectively (Supplementary Table 2)	Factor scores: General parenting, dysfunctional parenting (paternal side) and dysfunctional parenting (maternal side)
Friendship questionnaire (Goodyer et al., 1997; Van Harmelen et al., 2017)	This questionnaire assesses the number, availability and adequacy of friendships as well as self-disclosure and difficulties in friendships, and was adapted from previous research work. The individual questions were as follows: Are you happy with the number of friends you have? How often do you arrange to see friends other than at school, college or work? Do you feel that your friends understand you? Can you confide in your friends? Do your friends ever laugh at you or tease you in a hurtful way? Do people who aren’t your friends laugh at you or tease you in a hurtful way? Do you have arguments with your friends that upset you? Overall, how happy are you with your friendships? Item response options differed depending on the question but involved four to six possible responses (e.g. for the question ‘Do you feel that your friends understand you?’, the response options were most of the time, sometimes, not often or not at all, which were scored 0, 1, 2 or 3, respectively). EFA was used to determine the latent structure of the questionnaire (Supplementary Table 3)	Factor scores: Happiness/number of friends, confiding/understanding and teasing/arguments

BIS: Barratt Impulsiveness Scale; *DSM-5: Diagnostic and statistical manual of mental disorders* (5th ed.); CHI-T: Cambridge–Chicago Trait Compulsivity Scale; OBQ: Obsessive Beliefs Questionnaire; OCD: obsessive–compulsive disorder; OCPD: Obsessive–Compulsive Personality Disorder; MINI: Mini-International Neuropsychiatric Inventory; BBQ: Brunnsviken Brief Quality of life scale; APSD: Antisocial Process Screening Device; FAD: Family Assessment Device; PPQ: Positive Parenting Questionnaire; APQ: Alabama Parenting Questionnaire; MOPS: Measure of Parenting Style; NSPN: Neuroscience in Psychiatry Network; EFA: exploratory factor analysis.

Full details for the instrument citations are provided in the online supplement, due to space limitations.

The ICBC enquires about the presence of 33 types of impulsive and compulsive problem behaviours; for each type of behaviour, the individual endorses whether they and/or others think they have a problem with the behaviour, responding never, sometimes, often or always. The behaviours asked about by the ICBC are as follows: washing, smoking, feeling compelled to collect things, being overly cautious with money, re-arranging/ordering, shopping, list making, counting (e.g. money, tiles), grooming, idiosyncratic routines (performing a very personalised sequence of actions), repeating actions (over and over again), exercising, betting/gambling, hair pulling, lying, sexual activities/behaviours, alcohol consumption, planning (e.g. over-organising), illicit drug use, cleaning too much, verbal aggression, violence towards objects/properties, swearing, checking (e.g. locks, light switches), checking (e.g. yourself in the mirror), speed driving, medication use, physical aggression, social networking (e.g. Facebook, Twitter, Google+, MySpace), applying rules, purposeful self-injury (i.e. not accidental), re-writing/re-reading and tattooing. Previous work found the scale to have sound psychometric properties (Guo et al., 2017).

### Data analysis

All data analyses were conducted using JMP Pro Version 13.2.0 (SAS Institute, Inc.) and Mplus 7.2 (Muthén and Muthén, 1998–2012). The latent factor structure of the ICBC (the outcome instrument of interest) was examined using confirmatory factor analysis (CFA) of the covariance matrix using the weighted least squares means and variance (WLSMV) adjusted estimator and theta parameterisation (Muthén et al., 1997; Muthén and Muthén, 1998–2012). Based on prior studies and existing conceptual frameworks (Chamberlain et al., 2017; Guo et al., 2017; Reise, 2012), we evaluated a correlated two-factor model (two latent factors, i.e. impulsivity and compulsivity, correlated with each other), an orthogonal two-factor model (two latent factors, i.e. impulsivity and compulsivity, not correlated with each other), a one-factor model (one latent factor, corresponding to disinhibition) and a bi-factor model (two latent factors, i.e. impulsivity and compulsivity, plus a general factor). Post hoc model fitting was conducted by freeing theoretically plausible error covariances for estimation one at a time with reference to the highest modification index, and these were corrected for significance using the Benjamini–Hochberg False Discovery Rate (.05). Test of exact model fit using the chi-square test statistic (χ^2^) is overly sensitive to minor model misspecification in large sample sizes (Kline, 2016). Model fit was therefore evaluated using a combination of comparative fit indices, including the root mean square error of approximation (RMSEA; ε < .05 close approximate fit, ε = .05–.08 reasonable approximate fit, ε = >1.0 not close approximate fit), comparative fit index (CFI; >.90 reasonable fit, >.95 good fit) and the weighted root mean square residual (WRMR; <.950 good fit). We used a competing models approach, in which the comparative fit of several alternative models was evaluated to determine which provided the best representation of the covariances in the data.

Relationships between explanatory variables of interest and current impulsive–compulsive behaviours (latent scores on the ICBC) were investigated in two separate statistical models: the first examined baseline explanatory variables collected at study entry approximately (on average) 4 years prior, and the second model examined current explanatory variables (the broader range of impulsive and compulsive measures collected via the Internet in 2018). The statistical method of PLS was used. PLS is a versatile multivariate approach to data modelling that analyses relationships between exploratory (*X*) variables and outcome (*Y*) variables by means of fitting one or more latent components (Abdi and Williams, 2013). Unlike standard regression, PLS is robust to violations of normality assumptions and to item cross-correlations. Hence, PLS is ideally suited to the current dataset. Candidate explanatory (*X*) variables in the PLS models were summary scores from the questionnaires, and the outcome variables of interest (*Y*) were the ICBC latent scores.

For the model exploring baseline predictors of later impulsive and compulsive problem behaviours, the *X* variables of interest were as follows: age at baseline, gender, ethnic group, family education level (average years of education for the main caregiver), current or past mental health or behavioural problem (parent/guardian report), history of head trauma (parent/guardian report), history of any medical conditions (parent/guardian report), Barratt Impulsiveness Scale (BIS) scores, antisocial personality total scores, Padua Inventory obsessive–compulsive scores, general psychopathology (K10 scores, including anxiety/depression), parenting scores (general parenting, dysfunctional paternal parenting and dysfunctional maternal parenting) and friendship scores (happiness/number, confiding/understanding and teasing/arguments). For the model exploring current measures potentially associated with problem behaviours, *X* variables of interest were as follows: age, gender, education level, ethnic group, Barratt Impulsivity Scale scores, antisocial personality disorder traits (number of diagnostic criteria met), Padua Inventory obsessive–compulsive scores, Obsessive Beliefs Questionnaire (OBQ) scores, Cambridge–Chicago Trait Compulsivity Scale (CHI-T) compulsivity scores, Obsessive–Compulsive Personality Disorder (OCPD) traits (number of OCPD criteria met) and general psychopathology (presence of depression or anxiety symptoms).

For PLS, missing data points were imputed automatically by JMP software using mean substitution. The PLS models were fitted using leave-one-out cross-validation (non-linear iterative partial least squares [NIPALS] algorithm), and the optimal model was identified based on minimising predicted residual error sum of squares (PRESS). From each initial model, measures with a variable importance parameter (VIP) < 0.8 were excluded per convention (Cox and Gaudard, 2013). Explanatory (*X*) variables significantly contributing to the model (i.e. explaining significant variance in current compulsive and impulsive problem behaviours) were identified on the basis of 95% confidence intervals for bootstrap distribution of the standardised model coefficients not crossing zero (*N* = 1000 bootstraps; *p* < 0.05).

## Results

In total, 654 individuals completed the study. The mean (standard deviation) current age was 23.4 (3.2) years, and 34.7% were males. The current BIS-11 short mean total score was 16.4 (3.9) and the Padua Inventory mean score was 19.0 (19.1). Participants had returned their baseline data to NSPN on average 4.0 (1.3) years prior to participation in the current study and were of mean age 19.5 (3.1) years at baseline.

The majority of individuals (354 [53.2%]) endorsed having at least one problem behaviour in the preceding 12 months to some degree on the ICBC (Supplementary Table 1). A bi-factor model consisting of a general latent ‘Disinhibition’ factor with loadings from all 33 ICBC items and two specific factors capturing residual variance in 14 impulsivity (‘Impulsivity’) and 11 compulsivity items (‘Compulsivity’) provided the best overall fit to the ICBC data compared to three competing models (Table 2). The loadings of individual behaviours onto the latent factors are shown in Table 3, and the model had excellent fit (Reise, 2012). Bi-factor models have a tendency to provide superior fit compared to correlated factors and indirect hierarchical models because they have more parameters (Bonifay et al., 2017). Model diagnostics, including measures of construct replicability, reliability and unidimensionality, were conducted to ensure that the general and group factors were capturing meaningful covariances in the data and were not simply a product of overfitting (Rodriguez et al., 2016b). Construct replicability was evaluated by calculating the *H* index for the Disinhibition (.95), Impulsivity (.83) and Compulsivity (.69) factors, which ranges from 0 to 1 and quantifies the proportion of variance in a factor captured by its indicator variables (Hancock and Mueller, 2001). The results indicate that the strength of the standardised loadings for ICBC items on the latent factors were strong enough to suggest moderate–high replicability across studies using the same measures (Hancock and Mueller, 2001; Rodriguez et al., 2016b).

**Table 2.** Summary of fit statistics for the different competing confirmatory factor analysis models of the ICBC in the NSPN cohort.

	Model	*df*	χ^2^	*p*	RMSEA (90% CI)	CFI	WRMR
1	Correlated two-factor^a^	484	872.282	<0.001	.035 (.032–.039)	.960	1.146
2	Orthogonal two-factor^a^	485	3996.889	<0.001	.106 (.103–.109)	.643	3.507
3	One-factor^a^	485	1187.035	<0.001	.047 (.044–.051)	.929	1.403
4	Bi-factor^a^	461	647.001	<0.001	.025 (.020–.029)	.981	0.886

ICBC: Impulsive–Compulsive Behaviours Checklist; NSPN: Neuroscience in Psychiatry Network; FDR: False Discovery Rate; RMSEA: root mean square error of approximation; CI: confidence interval; CFI: comparative fit index; WRMR: weighted root mean square residual.

a Model included error covariances; all significant when corrected for multiple post hoc comparisons using the Benjamini–Hochberg FDR (*p* = 0.05), chi-square test statistic (χ^2^ > .05 exact fit), RMSEA (ε < .05 close approximate fit, ε = .05–.08 close approximate fit, ε = .08–1.0 reasonable approximate fit), CFI (>.90 reasonable fit, >.95 good fit) and WRMR (<.950 good fit).

**Table 3.** Standardised loading estimates for individual ICBC items on the disinhibition, impulsivity and compulsivity factors.

ICBC items	Disinhibition	Impulsivity	Compulsivity
1. Washing	.677		
2. Smoking	.141*	.585	
3. Collect	.519		
4. Money	.295		.375
5. Ordering	.626		.588
6. Shopping	.697		
7. List	.586		.496
8. Counting	.705		.385
9. Grooming	.790		
10. Routines	.674		.366
11. Repeating	.684		.234
12. Exercising	.629		
13. Betting	.312	.443	
14. Hair picking	.427		
15. Lying	.602	.405	
16. Sexual	.592	.498	
17. Alcohol	.379	.526	
18. Planning	.608		.451
19. Drug	.168*	.775	
20. Cleaning	.543		.422
21. Verbal	.625	.489	
22. Violence	.605	.504	
23. Swearing	.532	.470	
24. Checking locks	.515		.388
25. Checking mirror	.751		
26. Driving	.363	.201**	
27. Medication	.655	.214**	
28. Aggression	.637	.429	
29. Social	.616		
30. Rules	.636		.257
31. Injury	.474	.310	
32. Re-writing	.678		.295
33. Tattooing	.595	.437	

ICBC: Impulsive–Compulsive Behaviours Checklist.

All loading estimates were significant at *p* < 0.001, except where indicated.

* *p* < 0.05; **p < 0.01.

Calculation of the explained common variance (ECV) revealed that the Impulsivity and Compulsivity group factors collectively accounted for sufficient explained variance (31%) in the ICBC, in addition to the general Disinhibition factor (69%), to justify retaining them over a unidimensional model (Reise, 2012; Rodriguez et al., 2016a). Information functions generated for the 33 ICBC items loading on the general Disinhibition factor and were relatively distinct for different items (Supplementary Figure 1), suggesting that the general factor was not simply capturing item response bias or other forms of spurious variance (Bonifay et al., 2017). Factor score estimates were generated for the Disinhibition, Impulsivity and Compulsivity factors for use in subsequent analyses and exhibited weak bivariate correlations (Disinhibition with Impulsivity, *r* = .115, *p* = 0.004, and Compulsivity, *r* = .218, *p* < 0.001; Impulsivity with Compulsivity, *r* = –.177, *p* < 0.001). These low correlations indicated that the factor score estimates for each latent variable were not heavily contaminated by variance from the other two factors (Grice, 2001).

### Baseline measures associated with subsequent trans-diagnostic problem behaviours

**Figure 1.**
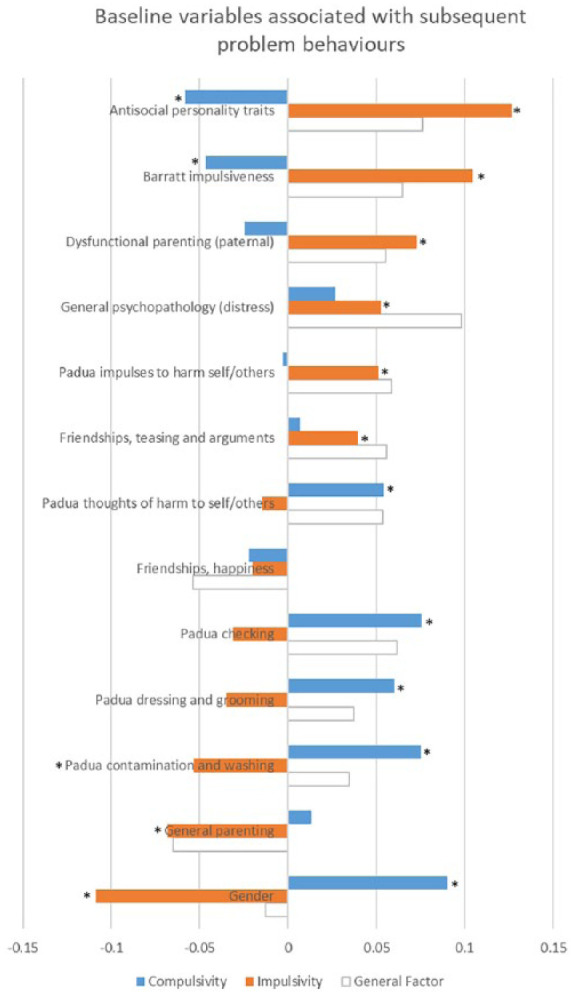
Standardised model coefficients for variables statistically explaining later problem behaviours (orange: impulsive problems; blue: compulsive problems). *p < 0.05, significant by rigorous statistical correction (bootstrap) for impulsive and compulsive problem behaviours. (For reference, model coefficients for the General Factor are shown in light grey outline; these were all significant by bootstrap except for gender [asterisks not shown].)

The optimal PLS model relating baseline variables to current impulsive–compulsive behaviours (ICBC latent scores) accounted for 37.4% of variation in the explanatory (*X*) measures and 11.6% of variation in later ICBC problem behaviour scores (*Y*). Variables that were important in the model (passing VIP threshold of 0.8) are shown in Figure 1. The following baseline variables were each significantly associated with higher subsequent impulsive problem behaviours: antisocial personality traits, impulsive traits (BIS), dysfunctional perceived parenting scores (both general parenting and paternal parenting scores), general psychopathology/distress (K10), male gender, impulses to harm self/others (Padua Inventory subscore) and teasing/arguments in friendships. Impulsive problem behaviours were also significantly predicted by previous lower contamination obsessions and washing compulsions (Padua Inventory subscore). Baseline variables that were significantly associated with higher subsequent compulsive problem behaviours were as follows: obsessional thoughts of harm to self/others (Padua Inventory subscore), compulsive checking (Padua subscore), dressing/grooming compulsions (Padua Inventory subscore), contamination obsessions and washing compulsions (Padua Inventory subscore) and female gender. Higher levels of compulsive problem behaviours were also significantly predicted by previous lower antisocial personality traits and lower impulsive traits (BIS). The following variables were statistically unimportant in the model and were excluded (VIP < 0.8): age at entry, ethnic group, family education level, current or past mental health or behavioural problem (parent/guardian report), history of head trauma (parent/guardian report), history of perinatal complications (parent/guardian report) and history of any medical conditions (parent/guardian report).

### Current measures associated with trans-diagnostic problem behaviours

**Figure 2.**
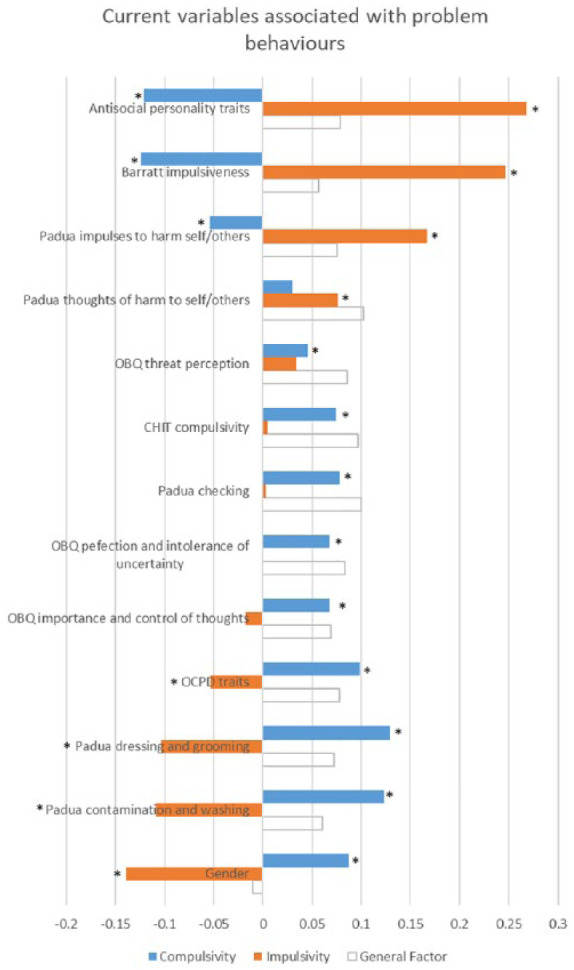
Standardised model coefficients for variables statistically explaining current problem behaviours (orange: impulsive problems; blue: compulsive problems). *p < 0.05, significant by rigorous statistical correction (bootstrap) for impulsive and compulsive problem behaviours. (For reference, model coefficients for the General Factor are shown in light grey outline; these were all significant by bootstrap except for gender [asterisks not shown].)

The optimal PLS model relating current variables to current impulsive–compulsive behaviours (ICBC latent scores) accounted for 48.0% of variation in the explanatory measures (*X*) and 32.7% of variation in ICBC problem behaviour scores (*Y*). Variables that were important in the model (passing VIP threshold of 0.8) are shown in Figure 2. The following measures were each significantly associated with higher impulsive problem behaviours: antisocial personality traits, impulsive traits (BIS), impulses to harm self/others (Padua Inventory subscore) and male gender. Higher impulsive problem behaviours were also significantly associated with fewer OCPD traits, lower dressing/grooming compulsions (Padua Inventory subscore) and lower contamination obsessions and washing compulsions (Padua Inventory subscore). The following measures were each significantly associated with higher compulsive problem behaviours: checking compulsions (Padua Inventory subscore), thoughts of harm to self/others (Padua Inventory subscore), dressing/grooming compulsions (Padua Inventory subscore), contam-ination obsessions and washing compulsions (Padua Inventory subscore), threat perception (OBQ), compulsive traits (CHI-T), perfection and intolerance of uncertainty (OBQ), importance and control of thoughts (OBQ), OCPD traits and female gender. Lower impulses to harm self/others (Padua Inventory subscore) was significantly associated with higher compulsive problem behaviours. The following variables were statistically unimportant in the model and were excluded (VIP < 0.8): age, ethnic group, education levels and general psychopathology (distress).

**Figure 3.**
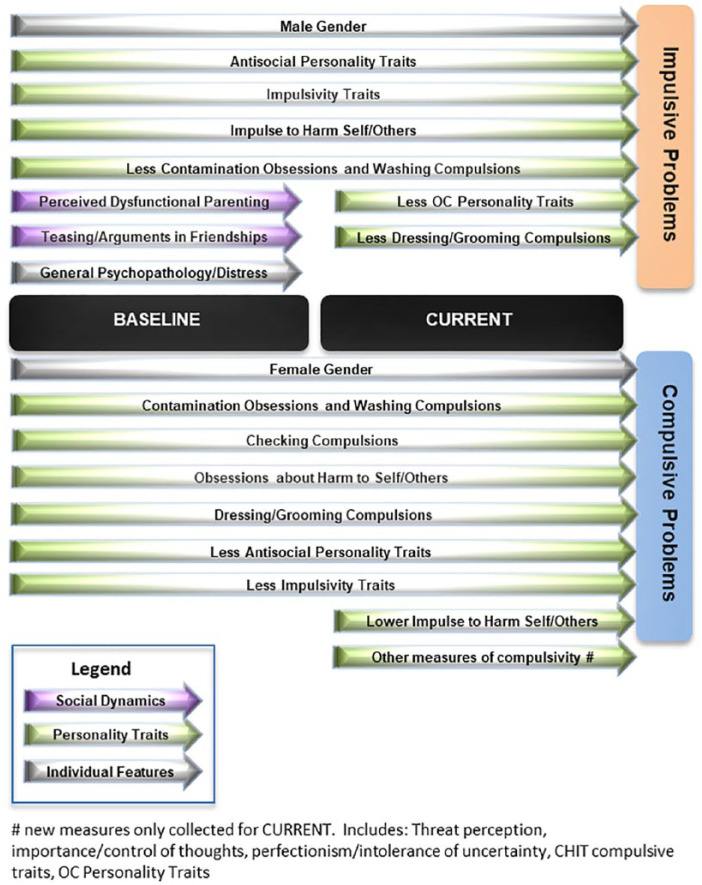
Variables significantly (*p* < 0.05, bootstrap in PLS models) mapping onto trans-diagnostic phenotypes of impulsive and compulsive problem behaviours. OC: obsessive–compulsive.

A conceptual schematic of the relationships between variables of interest and latent phenotype scores is provided in Figure 3.

## Discussion

This study examined latent phenotypes of impulsive and compulsive problem behaviours in young adults. We demonstrated that these behaviours were best conceptualised within a bi-factor model, which included two underlying latent phenotypes corresponding to impulsive and compulsive problems, respectively (Guo et al., 2017), as well as a general disinhibition factor. By modelling this additional general factor, we could quantify trans-diagnostic phenotypes that were separable and not due (for example) to common measurement variance (Reise, 2012). Predictors of the later emergence of these phenotypes were identified (Figure 3), incorporating relevant measures of personality, parenting and friendship experiences. The statistical approach used (PLS) allowed intrinsic control for co-relationships between the important explanatory variables of interest. We did not identify any significant effects of age or education levels on occurrence of problem impulsive or compulsive behaviours.

Personality-related questionnaires provide rich dimensional measures and are typically developed for use in normative settings, as well as in patient populations (Sanavio, 1988; Stanford et al., 2016). Personality measures here showed the clearest differentiation in statistically predicting later trans-diagnostic occurrence of impulsive and compulsive problem behaviours (Figure 3). Higher baseline scores on the Barratt Impulsivity Scale (Stanford et al., 2016) and the Antisocial Process Screening Device (Vitacco et al., 2003) each significantly predicted higher subsequent impulsivity scores derived from the ICBC (an independent instrument), and contrariwise, each of these two baseline personality scores significantly predicted lower subsequent compulsive problem behaviours.

As anticipated, most of the Padua Obsessive–Compulsive Inventory subscores (Sanavio, 1988) were associated with later emergence of compulsive but not impulsive problem behaviours, especially so for archetypal contamination obsessions and washing compulsions (for further discussion of Padua Inventory results, see Supplemental Material). We view scores on the Padua Inventory as personality traits in that they are measurable in the general population along a continuum. In fact, the instrument was originally developed for community use (Sanavio, 1988); this is in contrast to clinical measures of OCD, such as the Yale–Brown Obsessive Compulsive Scale (Y-BOCS), which are unsuitable for phenotyping research due to a high likelihood of zero score in those without formal OCD (Goodman et al., 1989).

While the above associations are largely expected, in view of the conceptual domains they encompass, the results also indicate important, differential contribution of earlier personality traits known to be partly heritable (Jonnal et al., 2000; Niv et al., 2012; Tuvblad et al., 2014) to the ultimate manifestation of an extensive array of 33 adult problem behaviours. This pattern of association for impulsive, antisocial and obsessive–compulsive traits was also replicated cross-sectionally, but extending to a broader range of compulsivity instruments not originally included at baseline in the NSPN cohort (Figure 3).

It is well established that among environmental factors relevant to later risk of mental health problems, parenting and friendship networks are particularly important in young people, along with general distress. For example, family and friendship support during early adolescence negatively predicted risk of later adolescent depressive symptoms arising as a function of childhood adversity (Van Harmelen et al., 2016). Here, we show that worse perceived general parenting, higher levels of perceived dysfunctional parenting (reported by the study participant in relation to their father) and higher levels of general distress (K10 instrument) were specifically associated with later emergence of impulsive but not compulsive problems (Figure 3). Furthermore, participants’ earlier experiences of teasing and arguments within their friendship network, and not other aspects of friendship (as measured), were also significantly associated with later impulsive but not compulsive problem behaviours. Family functioning is an important determinant of specific types of impulsive problems (such as non-suicidal self-injury; Cassels et al., 2018). Positive relationships between dysfunctional parenting (especially harshness and psychological control) and externalising symptoms have been shown in prior meta-analysis, in young people (Pinquart, 2017). In a longitudinal population-based study of adopted and non-adopted adolescents, parent–child conflict was associated with subsequent impulsive (‘acting-out’) behaviours (Klahr et al., 2011a, 2011b).

We found a dissociation in the influence of gender on impulsive and compulsive trans-diagnostic phenotypes: male gender was significantly associated with impulsive problem behaviours (viewed trans-diagnostically), whereas female gender was significantly associated with compulsive problem behaviours. Impulsive problems (such as aggression and criminality) are more common in males in much of the literature (Chamorro et al., 2012), findings which may be due to males having higher sensation seeking and risk-taking (Cross et al., 2011). Several previous cross-sectional studies found that female gender is associated with higher obsessive–compulsive traits on the Padua Inventory (Chamberlain et al., 2016; Sanavio, 1988). These results hint at sexually divergent processes in the development of impulsive versus compulsive problems, which are not due (for example) to confounding gender differences in personality traits, parenting or friendships, which were controlled for in the statistical modelling. Our data also accord well with findings from literature in other areas, particularly internalising–externalising studies in young people into adulthood, which commonly report a similar gender discrepancy (Wilhelm, 2014).

The bi-factor modelling approach is increasingly adopted in psychiatry research (Caspi et al., 2014; Castellanos-Ryan et al., 2016; Hankin et al., 2016; Patalay et al., 2015; Stochl et al., 2015). Here, we demonstrate that a bi-factor model yielded superior fit across all metrics for the fractionation of trans-diagnostic phenotypes of impulsive and compulsive problem behaviours. To measure the phenotypes of interest, we used responses from the ICBC, which examined a broad range of 33 behaviours in a single, convenient questionnaire. The nature of the general latent factor, within the bi-factor model, itself is open to interpretation, as in other studies. The general factor was associated with all study measures except gender, suggesting that it may relate to general disinhibition (general tendencies towards failure to suppress behaviours). We believe it unlikely that this general factor reflects only common response bias because its relationship with individual ICBC items was variable. It is intended to examine biological substrates of this general disinhibition factor in future work.

There are several limitations in relation to this study. Rigorous demonstration of causality has multiple scientific requirements and cannot be shown definitively within a study design as deployed here. Nonetheless, impulsive, dissocial and compulsive traits bore strong relationships with their respective expected problem behaviours (measured using a separate questionnaire) both longitudinally and cross-sectionally, showing a sustained and meaningful association. The original recruitment methodology for NSPN was designed to be epidemiologically representative (i.e. stratified based on census statistics; Kiddle et al., 2017). However, the current study participants provided data in 2018 and thus may not be fully representative of the original NSPN cohort. Our current sample had mean impulsive and compulsive scores (Barratt Impulsivity Scale and Padua Inventory) similar to those reported in previous normative studies (Burns et al., 1996; Steinberg et al., 2013). We measured relatively ‘high level’ trans-diagnostic phenotypes closely allied to overt problems, but the phenotypic approach should ultimately be applied to a range of measures incorporating also cognitive and biological (genetic, imaging) parameters. Finally, the best fit statistical model for data is contingent on the particular nature and range of measures used; because a given model offers a superior fit, it does not follow that other models have no utility.

In summary, impulsive and compulsive problem behaviours were fractionated trans-diagnostically using a bi-factor model, which yielded the best fit versus other alternative models suggested by the extant literature. Significant baseline predictors of later problem impulsive and compulsive trans-diagnostic phenotypes were identified, using a powerful statistical technique well suited to large datasets where items are likely to be correlated. Personality traits were important determinants of impulsive and compulsive problem behaviours, whereas parenting and friendship experiences significantly predicted impulsive problems specifically, and gender specific effects were found (male gender, higher impulsive problems; female gender, higher compulsive problems). This study highlights the potential utility of the trans-diagnostic approach in psychiatry, as applied to impulsivity and compulsivity. The heritability and biological substrates of latent impulsivity, compulsivity and general model factors merit exploration. For example, if the trans-diagnostic phenotype of compulsivity has higher heritability than impulsivity, this may account for the relatively lower impact of the earlier social milieu on the former. The finding that aspects of parenting and friendships differentially relate to impulsive as opposed to compulsive problems may have implications for tailoring early interventions and targeting vulnerable individuals to avert the development of later problematic behaviours. In particular, such trans-diagnostic approaches can be used to identify candidate risk factors for a broad range of psychiatric symptoms. These risk factors (such as elements of personality, friendship support and parenting) could in future be targeted for modification in people at risk of impulsive and compulsive problems, in order to avert the development of these impairing pathologies. This would potentially shift the emphasis in psychiatry more towards prevention (Furber et al., 2017), as well as treatment – a shift that has been deployed with success in other areas of healthcare and in other areas of psychiatry outside the impulsive–compulsive sphere.

## Supplemental Material

supplementary_file – Supplemental material for Fractionation of impulsive and compulsive trans-diagnostic phenotypes and their longitudinal associations Supplemental material, supplementary_file for Fractionation of impulsive and compulsive trans-diagnostic phenotypes and their longitudinal associations by Samuel R Chamberlain, Jeggan Tiego, Leonardo F Fontenelle, Roxanne Hook, Linden Parkes, Rebecca Segrave, Tobias U Hauser, Ray J Dolan, Ian M Goodyer, Ed Bullmore, Jon E Grant and Murat Yücel in Australian & New Zealand Journal of Psychiatry
