# Supplementary material for: Fractionation of impulsive and compulsive trans-diagnostic phenotypes and their longitudinal associations
Source: Aust N Z J Psychiatry. 2019 Apr 19;53(9):896–907. doi: 10.1177/0004867419844325 (PMC6724459; doi:10.1177/0004867419844325)
Supplement: supplementary_file – Supplemental material for Fractionation of impulsive and compulsive trans-diagnostic phenotypes and their longitudinal associations [file supplementary_file.pdf]

Supplementary Online File – Chamberlain et al.

Supplementary Table 1. Number and percentage of sample endorsing each problematic behaviour, ranked by highest to lowest. For original item descriptions and validation see (Guo et al., 2017).

	Problematic (often/always)	
ICBC Scale item (behaviour)	N	%
impcomp_7_list	97	14.8
impcomp_5_ordering	88	13.5
impcomp_4_money	85	13.0
impcomp_6_shopping	81	12.4
impcomp_18_planning	73	11.2
impcomp_23_swearing	73	11.2
impcomp_29_social_networking	72	11.0
impcomp_24_checkinglocks	68	10.4
impcomp_8_counting	61	9.3
impcomp_12_exercising	57	8.7
impcomp_3_collect	56	8.6
impcomp_25_checkingmirror	56	8.6
impcomp_32_rewriting	53	8.1
impcomp_10_routines	51	7.8
impcomp_9_grooming	45	6.9
impcomp_1_washing	41	6.3
impcomp_14_hairpicking	39	6.0
impcomp_11_repeating	37	5.7
impcomp_17_alcohol	35	5.4
impcomp_30_rules	34	5.2
impcomp_20_cleaning	32	4.9
impcomp_16_sexual	31	4.7
impcomp_15_lying	26	4.0
impcomp_21_verbal	24	3.7
impcomp_31_injury	18	2.8
impcomp_2_smoking	17	2.6
impcomp_26_driving	15	2.3
impcomp_19_drug	12	1.8
impcomp_33_tattooing	12	1.8
impcomp_27_medication	11	1.7
impcomp_22_violence	10	1.5
impcomp_28_aggression	10	1.5
impcomp_13_betting	8	1.2

Supplementary Table 2. Factor analysis for Family measures; baseline FAD, PPQ, and MOPs. Varimax rotation. Factor analysis yielded an optimal three-factor solution corresponding broadly to general parenting, paternal parenting, and maternal parenting.

	Factor 1	Factor 2	Factor 3
fad_01_planning_activities_B1	-0.547238	0.208334	0.076752
fad_02_cant_talk_sadness_B1	-0.672641	0.129103	0.058860
fad_03_feel_accepted_B1	0.472165	-0.264937	-0.080512
fad_04_dont_get_along_B1	-0.565752	0.245882	0.180767
fad_05_express_feelings_B1	0.674635	-0.133244	-0.066200
fad_06_crisis_support_B1	0.601494	-0.152014	-0.031224
fad_07_avoid_fears_B1	-0.526606	0.081896	0.043102
fad_08_solve_problems_B1	0.558286	-0.207280	-0.153954
fad_09_confide_each_other_B1	0.662436	-0.093072	-0.026798
fad_10_bad_feelings_B1	-0.471003	0.298365	0.131157
fad_11_accepted_B1	0.507883	-0.241655	-0.111908
fad_12_decisions_problem_B1	-0.487254	0.248221	0.099861
apq_01_good_job_B1	0.609592	-0.149168	-0.228817
apq_02_threaten_punish_B1	-0.265094	0.180640	0.111741
apq_03_fails_note_B1	-0.288870	0.102757	0.081039
apq_04_games_B1	0.625299	-0.150003	-0.195314
apq_05_talk_out_punish_B1	-0.080303	0.068991	-0.020814
apq_06_ask_school_B1	0.516450	-0.114427	-0.270524
apq_07_stays_out_B1	-0.144916	0.097129	0.058304
apq_08_homework_B1	0.476666	-0.112805	-0.128946
apq_09_compliment_B1	0.646527	-0.145398	-0.287235
apq_10_praise_B1	0.549897	-0.143812	-0.184160
apq_11_out_friends_B1	-0.402493	0.087886	0.107516
apq_12_punish_early_B1	-0.045177	0.070352	0.012837
apq_13_spank_B1	-0.143259	0.230561	0.241285
apq_14_slap_B1	-0.235086	0.310520	0.276571
apq_15_hit_B1	-0.168838	0.220696	0.208459
mops_mum01_overprotective_B1	-0.117161	0.121324	-0.139883
mops_mum02_abusive_B1	-0.279530	0.165638	0.569752
mops_mum03_overcontrolling_B1	-0.235632	0.196838	0.218999
mops_mum04_guilty_B1	-0.250222	0.269687	0.456317
mops_mum05_ignored_B1	-0.169700	0.186528	0.677657
mops_mum06_critical_B1	-0.296096	0.218219	0.409755
mops_mum07_unpredictable_B1	-0.180801	0.158012	0.603880
mops_mum08_uncaring_B1	-0.146691	0.018113	0.829674
mops_mum09_violent_B1	-0.213518	0.177831	0.596283
mops_mum10_rejecting_B1	-0.105363	0.054073	0.773359
mops_mum11_left_B1	-0.232416	0.128328	0.515551
mops_mum12_forget_B1	-0.113307	0.004932	0.707009
mops_mum13_uninterested_B1	-0.150325	-0.035921	0.722987
mops_mum14_danger_B1	-0.071321	0.137109	0.607757
mops_mum15_unsafe_B1	-0.101437	0.066390	0.649114
mops_dad01_overprotective_B1	-0.118675	-0.095268	-0.023414
mops_dad02_abusive_B1	-0.302794	0.615893	0.068391
mops_dad03_overcontrolling_B1	-0.296815	0.281799	0.090779
mops_dad04_guilty_B1	-0.291750	0.556320	0.129004
mops_dad05_ignored_B1	-0.193290	0.745416	0.157472
mops_dad06_critical_B1	-0.249017	0.549219	0.077189
mops_dad07_unpredictable_B1	-0.252191	0.666023	0.108111
mops_dad08_uncaring_B1	-0.150943	0.810779	0.157088
mops_dad09_violent_B1	-0.228340	0.563792	0.040966
mops_dad10_rejecting_B1	-0.123108	0.796905	0.197000
mops_dad11_left_B1	-0.199347	0.630235	0.172926
mops_dad12_forget_B1	-0.113729	0.744063	0.182330
mops_dad13_uninterested_B1	-0.168528	0.738255	0.148740
mops_dad14_danger_B1	-0.117250	0.759869	0.080733
mops_dad15_unsafe_B1	-0.154001	0.748807	0.086786
ppq_01_quality_time_B1	0.659200	-0.111140	-0.133581
ppq_02_attend_school_B1	0.553836	-0.205185	-0.264559

	Factor 1	Factor 2	Factor 3
ppq_03_affection_B1	0.647323	-0.151684	-0.187810
ppq_04_get_me_B1	0.490038	-0.241933	-0.234575
ppq_05_comforted_B1	0.741944	-0.218714	-0.203940
ppq_06_angry_listen_B1	0.739782	-0.139884	-0.176330
ppq_07_praised_B1	0.676847	-0.185377	-0.305175
ppq_08_ideas_encouraged_B1	0.702899	-0.208536	-0.279036
ppq_09_priority_B1	0.689143	-0.201396	-0.306553
ppq_10_loved_B1	0.656038	-0.259801	-0.354993
ppq_11_listened_to_B1	0.772309	-0.228558	-0.249767
ppq_12_contact_B1	0.546596	-0.206974	-0.300136
ppq_13_home_safe_B1	0.394675	-0.388621	-0.413940
ppq_14_opinions_valued_B1	0.755413	-0.200207	-0.242450
ppq_15_talked_important_B1	0.790570	-0.128205	-0.215853
ppq_16_privacy_respected_B1	0.569107	-0.247863	-0.173495
ppq_17_friends_welcomed_B1	0.386843	-0.231163	-0.262560
ppq_18_clothes_B1	0.301213	-0.194910	-0.383426
ppq_19_pocket_money_B1	0.336293	-0.104612	-0.150965
ppq_20_ask_B1	0.541811	-0.219459	-0.250330
ppq_21_achieve_B1	0.405041	-0.104150	-0.433269
ppq_22_cared_unwell_B1	0.387813	-0.184888	-0.375585
ppq_23_skills_B1	0.608445	-0.208298	-0.298424
ppq_24_advice_B1	0.693763	-0.204428	-0.261923
ppq_25_learn_school_B1	0.349574	-0.132913	-0.427536
ppq_26_progress_B1	0.439963	-0.147804	-0.396667

Supplementary Table 3. Factor analysis for baseline friendship questionnaire. Varimax rotation. Factor analysis yielded an optimal three-factor solution corresponding broadly to number of friend and happiness; confiding and understanding; and teasing/arguments.

	Factor 1	Factor 2	Factor 3
fq_01_number_friends_B1	0.234507	0.741151	-0.111058
fq_02_see_friends_B1	0.363220	0.388374	0.133014
fq_03_friends_understand_B1	0.809284	0.318706	-0.220105
fq_04_confide_in_friends_B1	0.665423	0.319904	-0.152303
fq_05_friends_tease_B1	-0.057429	-0.026811	0.666492
fq_06_not_friends_tease_B1	-0.113377	-0.019231	0.524172
fq_07_arguments_B1	-0.039992	-0.114816	0.670419
fq_08_overall_happy_B1	0.414031	0.713689	-0.183807

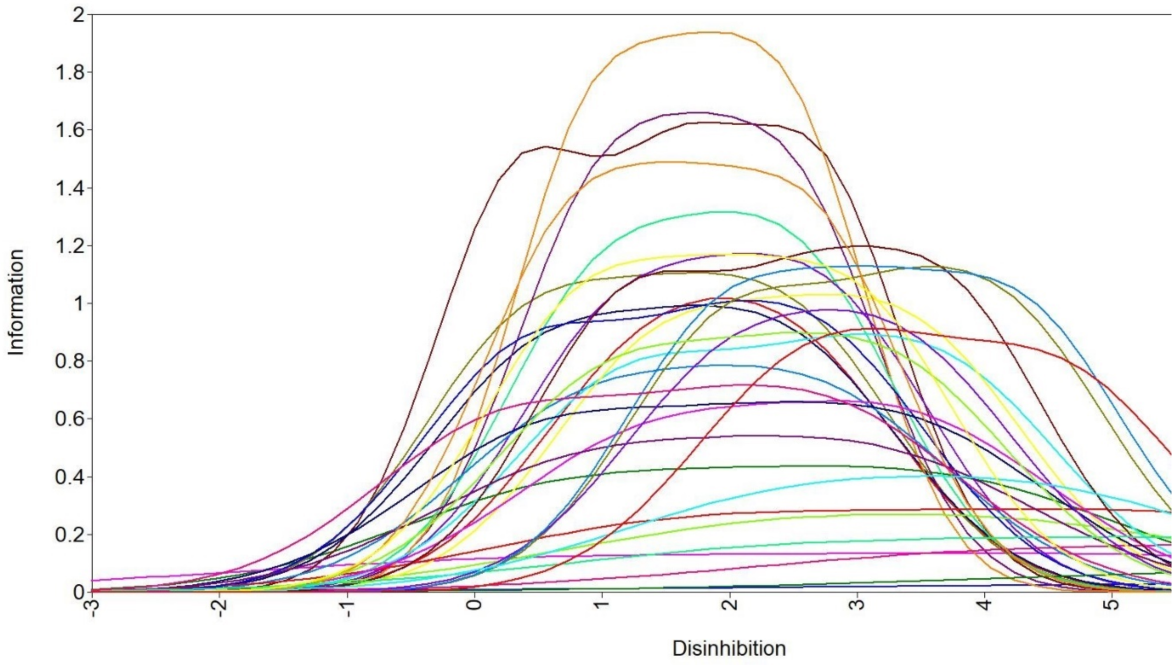

Supplementary Figure 1. Item information functions for the 33 ICBC items in relation to the latent trait Disinhibition (θ). The information functions were distinct for different items, suggesting that the general Disinhibition factor from the bi-factor model of the ICBC was not attributable to response bias.

Additional Discussion regarding OC traits on the Padua Inventory

As anticipated, most of the Padua obsessive-compulsive inventory subscores (Sanavio, 1988) were associated with later emergence of compulsive but not impulsive problem behaviours, especially so for archetypal contamination obsessions and washing compulsions. The one exception here was the 'impulses to harm self/others' Padua subscore, which was associated with impulsive problems, indicating that items originally designed to measure compulsivity in fact are endorsed by impulsive individuals, with implications for possible future revisions of this instrument. We view scores on the Padua inventory personality traits in that they are measurable in the general population along a continuum. In fact the instrument was originally developed for community use (Sanavio, 1988); this is in contrast to clinical measures of OCD, such as the Yale-Brown Obsessive Compulsive Scale, which are unsuitable for phenotyping research due to a high likelihood of zero score in those without formal OCD.

References

Abdi H and Williams LJ. (2013) Partial least squares methods: partial least squares correlation and partial least square regression. *Methods Mol Biol* 930: 549-579.

American Psychiatric Association. (2013) *Diagnostic and statistical manual of mental disorders (5th ed.) (DSM-5),* Arlington, VA: American Psychiatric Publishing.

Bagozzi RP and Yi Y. (2012) Specification, evaluation, and interpretation of structural equation models. *Journal of the academy of marketing science* 40: 8 - 34.

Benjamini Y and Hochberg Y. (1995) Controlling the False Discovery Rate: A Practical and Powerful Approach to Multiple Testing. *Journal of the Royal Statistical Society. Series B (Methodological)* 57: 289-300.

Bentler PM and Bonett DG. (1980) Significance tests and goodness of fit in the analysis of covariance structures. *Psychological Bulletin* 88: 588 - 606.

Bonifay W, Lane SP and Reise SP. (2017) Three concerns with applying a bifactor model as a structure of psychopathology. *Clinical Psychological Science* 5: 184-186.

Browne MW and Cudeck R. (1993) Alternative ways of assessing model fit. In: Bollen KA and Long JS (eds) *Testing Structural Equation Models.* Newbury Park, CA: Sage, 136 -162

Burns GL. (1995) *Padua Inventory-Washington State University Revision.* : Pullman, WA. (Available from G. Leonard Burns, Department of Psychology, Washington State University, Pullman, WA 99164-4820).

Burns GL, Keortge SG, Formea GM, et al. (1996) Revision of the Padua Inventory of obsessive compulsive disorder symptoms: distinctions between worry, obsessions, and compulsions. *Behav Res Ther* 34: 163-173.

Chamberlain SR and Grant JE. (2018) Initial Validation of a Trans-diagnostic Compulsivity Questionnaire: The Cambridge-Chicago Compulsivity Trait Scale. *CNS Spectrums* In press.

Chamberlain SR, Stochl J, Redden SA, et al. (2017) Latent traits of impulsivity and compulsivity: toward dimensional psychiatry. *Psychol Med* 48: 810-821.

Cox I and Gaudard M. (2013) *Discovering Partial Least Squares with JMP,* Cary, North Carolina, USA: SAS Institute Inc.

Cramer RD. (1993) Partial Least Squares (PLS): Its strengths and limitations. *Perspectives in Drug Discovery and Design* 1: 269-278.

Cribbie RA. (2007) Multiplicity control in structural equation modeling. *Structural equation modeling* 14: 98-112.

Elgar FJ, Waschbusch DA, Dadds MR, et al. (2007) Development and Validation of a Short Form of the Alabama Parenting Questionnaire. *Journal of Child anf Family Studies* 16: 243-259.

Frick PJ, O'Brien BS, Wootton JM, et al. (1994) Psychopathy and conduct problems in children. *J Abnorm Psychol* 103: 700-707.

Garthwaite PH. (1994) An Interpretation of Partial Least Squares. *Journal of the American Statistical Association* 89: 122-127.

Goodyer IM, Herbert J, Tamplin A, et al. (1997) Short-term outcome of major depression: II. Life events, family dysfunction, and friendship difficulties as predictors of persistent disorder. *J Am Acad Child Adolesc Psychiatry* 36: 474-480.

Grice JW. (2001) Computing and evaluating factor scores. *Psychological methods* 6: 430-450.

Guo K, Youssef GJ, Dawson A, et al. (2017) A psychometric validation study of the Impulsive-Compulsive Behaviours Checklist: A transdiagnostic tool for addictive and compulsive behaviours. *Addict Behav* 67: 26-33.

Hancock GR and Mueller RO. (2001) Rethinking construct reliability within latent variable systems. In: Cudeck R, du Toit S and Sorbom D (eds) *Structural equation modeling: Present and future - A festchrift in honor of Karl Joreskog.* Lincolnwood, IL: Scientific Software International, 195-216.

Höskuldsson A. (1988) PLS regression methods. *Journal of Chemometrics* 2: 211-228.

Kessler RC, Andrews G, Colpe LJ, et al. (2002) Short screening scales to monitor population prevalences and trends in non-specific psychological distress. *Psychol Med* 32: 959-976.

Kline RB. (2016) *Principles and practice of structural equation modeling,* New York: The Guilford Press.
Lindner P, Frykheden O, Forsstrom D, et al. (2016) The Brunnsviken Brief Quality of Life Scale (BBQ): Development and Psychometric Evaluation. *Cogn Behav Ther* 45: 182-195.
Marsh HW, Hau KT and Wen Z. (2004) In search of golden rules: Comment on hypothesis-testing approaches to setting cutoff values for fit indexes and dangers in overgeneralizing Hu and Bentler's (1999) findings. *Structural equation modeling* 11: 320-341.
Menzies L, Achard S, Chamberlain SR, et al. (2007) Neurocognitive endophenotypes of obsessive-compulsive disorder. *Brain* 130: 3223-3236.
Moulding R, Anglim J, Nedeljkovic M, et al. (2011) The Obsessive Beliefs Questionnaire (OBQ): examination in nonclinical samples and development of a short version. *Assessment* 18: 357-374.
Munoz LC and Frick PJ. (2007) The reliability, stability, and predictive utility of the self-report version of the Antisocial Process Screening Device. *Scand J Psychol* 48: 299-312.
Muthén B, du Toit SHC and Spisic D. (1997) Robust inference using weighted least squares and quadratic estimating equations in latent variable modeling with categorical and continuous outcomes. *Conditionallly accepted for publication in Psychometrika.*
Muthén LK and Muthén BO. (1998 - 2012) *Mplus user's guide.,* Los Angeles, CA: Muthén & Muthén.
Parker G, Roussos J, Hadzi-Pavlovic D, et al. (1997) The development of a refined measure of dysfunctional parenting and assessment of its relevance in patients with affective disorders. *Psychol Med* 27: 1193-1203.
Patton JH, Stanford MS and Barratt ES. (1995) Factor structure of the Barratt impulsiveness scale. *J. Clin. Psychol.* 51: 768-774.
Reise SP. (2012a) Invited Paper: The Rediscovery of Bifactor Measurement Models. *Multivariate Behav Res* 47: 667-696.
Reise SP. (2012b) The rediscovery of bifactor measurement models. *Multivariate Behavioral Research* 47: 667 - 696.
Ridenour TA, Daley JG and Reich W. (1999) Factor analyses of the family assessment device. *Fam Process* 38: 497-510.
Robbins TW, Gillan CM, Smith DG, et al. (2012) Neurocognitive endophenotypes of impulsivity and compulsivity: Towards dimensional psychiatry. *Trends in Cognitive Sciences* 16: 81 - 91.
Rodriguez A, Reise SP and Haviland MG. (2016a) Applying bifactor statistical indices in the evaluation of psychological measures. *J Pers Assess* 98: 223-237.
Rodriguez A, Reise SP and Haviland MG. (2016b) Evaluating bifactor models: Calculating and interpreting statistical indices. *Psychol Methods* 21: 137-150.
Sanavio E. (1988) Obsessions and compulsions: the Padua Inventory. *Behav Res Ther* 26: 169-177.
Sheehan DV, Lecrubier Y, Sheehan KH, et al. (1998) The Mini-International Neuropsychiatric Interview (M.I.N.I.): the development and validation of a structured diagnostic psychiatric interview for DSM-IV and ICD-10. *J Clin Psychiatry* 59 Suppl 20: 22-33;quiz 34-57.
Silvia ESM and MacCallum RC. (1988) Some factors affecting the success of specification searches in covariance structure modeling. *Multivariate Behavioral Research* 23: 297 - 326.
Stanford MS, Mathias CW, Dougherty DM, et al. (2016) Fifty years of the Barratt Impulsiveness Scale: An update and review. *Personality and Individual Differences* 47: 385-395.
Steinberg L, Sharp C, Stanford MS, et al. (2013) New tricks for an old measure: the development of the Barratt Impulsiveness Scale-Brief (BIS-Brief). *Psychol Assess* 25: 216-226.
Yu C-Y. (2002) Evaluating cutoff criteria of model fit indices for latent variable models with binary and continuous outcomes. University of California Los Angeles.

Additional References for Study Instruments in Main Manuscript

American Psychiatric Association (2013). Diagnostic and statistical manual of mental disorders (5th ed.) (DSM-5). Arlington, VA, American Psychiatric Publishing.

Burns, G. L. (1995). Padua Inventory-Washington State University Revision. , Pullman, WA. (Available from G. Leonard Burns, Department of Psychology, Washington State University, Pullman, WA 99164-4820).

Chamberlain, S. R. and J. E. Grant (2018). "Initial Validation of a Trans-diagnostic Compulsivity Questionnaire: The Cambridge-Chicago Compulsivity Trait Scale." CNS Spectrums In press.

Elgar, F. J., D. A. Waschbusch, M. R. Dadds and N. Sigvaldason (2007). "Development and Validation of a Short Form of the Alabama Parenting Questionnaire." Journal of Child anf Family Studies 16(2): 243-259.

Goodyer, I. M., J. Herbert, A. Tamplin, S. M. Secher and J. Pearson (1997). "Short-term outcome of major depression: II. Life events, family dysfunction, and friendship difficulties as predictors of persistent disorder." J Am Acad Child Adolesc Psychiatry 36(4): 474-480.

Kessler, R. C., G. Andrews, L. J. Colpe, E. Hiripi, D. K. Mroczek, S. L. Normand, E. E. Walters and A. M. Zaslavsky (2002). "Short screening scales to monitor population prevalences and trends in non-specific psychological distress." Psychol Med 32(6): 959-976.

Lindner, P., O. Frykheden, D. Forsstrom, E. Andersson, B. Ljotsson, E. Hedman, G. Andersson and P. Carlbring (2016). "The Brunnsviken Brief Quality of Life Scale (BBQ): Development and Psychometric Evaluation." Cogn Behav Ther 45(3): 182-195.

Moulding, R., J. Anglim, M. Nedeljkovic, G. Doron, M. Kyrios and A. Ayalon (2011). "The Obsessive Beliefs Questionnaire (OBQ): examination in nonclinical samples and development of a short version." Assessment 18(3): 357-374.

Munoz, L. C. and P. J. Frick (2007). "The reliability, stability, and predictive utility of the self-report version of the Antisocial Process Screening Device." Scand J Psychol 48(4): 299-312.

Parker, G., J. Roussos, D. Hadzi-Pavlovic, P. Mitchell, K. Wilhelm and M. P. Austin (1997). "The development of a refined measure of dysfunctional parenting and assessment of its relevance in patients with affective disorders." Psychol Med 27(5): 1193-1203.

Patton, J. H., M. S. Stanford and E. S. Barratt (1995). "Factor structure of the Barratt impulsiveness scale." J. Clin. Psychol. 51(6): 768-774.

Ridenour, T. A., J. G. Daley and W. Reich (1999). "Factor analyses of the family assessment device." Fam Process 38(4): 497-510.

Sanavio, E. (1988). "Obsessions and compulsions: the Padua Inventory." Behav Res Ther 26(2): 169-177.

Sheehan, D. V., Y. Lecrubier, K. H. Sheehan, P. Amorim, J. Janavs, E. Weiller, T. Hergueta, R. Baker and G. C. Dunbar (1998). "The Mini-International Neuropsychiatric Interview (M.I.N.I.): the development and validation of a structured diagnostic psychiatric interview for DSM-IV and ICD-10." J Clin Psychiatry 59 Suppl 20: 22-33;quiz 34-57.

Stanford, M. S., C. W. Mathias, D. M. Dougherty, S. L. Lake, N. E. Anderson and J. H. Patton (2016). "Fifty years of the Barratt Impulsiveness Scale: An update and review." Personality and Individual Differences 47(5): 385-395.

Steinberg, L., C. Sharp, M. S. Stanford and A. T. Tharp (2013). "New tricks for an old measure: the development of the Barratt Impulsiveness Scale-Brief (BIS-Brief)." Psychol Assess 25(1): 216-226.

van Harmelen, A. L., R. A. Kievit, K. Ioannidis, S. Neufeld, P. B. Jones, E. Bullmore, R. Dolan, N. Consortium, P. Fonagy and I. Goodyer (2017). "Adolescent friendships predict later resilient functioning across psychosocial domains in a healthy community cohort." Psychol Med 47(13): 2312-2322.
